# Evaluation of sarcopenia and myosteatosis to determine the impact on mortality after emergency laparotomy

**DOI:** 10.1093/bjsopen/zraf092

**Published:** 2025-08-05

**Authors:** Richard P T Evans, Dimit Raveshia, Mei Sien Liew, Anna Jackowski, Aaron Kisiel, Ewen A Griffiths, Benjamin H L Tan

**Affiliations:** Department of Upper Gastrointestinal Surgery, Queen Elizabeth Hospital, Birmingham, UK; Institute of Immunology and Immunotherapy, University of Birmingham, Birmingham, UK; Department of Upper Gastrointestinal Surgery, Queen Elizabeth Hospital, Birmingham, UK; Department of Upper Gastrointestinal Surgery, Queen Elizabeth Hospital, Birmingham, UK; Department of Upper Gastrointestinal Surgery, Queen Elizabeth Hospital, Birmingham, UK; Department of Upper Gastrointestinal Surgery, Queen Elizabeth Hospital, Birmingham, UK; Department of Upper Gastrointestinal Surgery, Queen Elizabeth Hospital, Birmingham, UK; Institute of Immunology and Immunotherapy, University of Birmingham, Birmingham, UK; Department of Upper Gastrointestinal Surgery, Queen Elizabeth Hospital, Birmingham, UK; Institute of Immunology and Immunotherapy, University of Birmingham, Birmingham, UK

**Keywords:** sarcopenia, myosteatosis, emergency laparotomy, mortality, morbidity

## Abstract

**Background:**

Emergency laparotomy is performed for a wide range of life-threatening conditions and is associated with significant morbidity and mortality. Risk prediction models facilitate accurate communication of operative risk with patients and relatives, in addition to benchmarking unit outcomes. Greater understanding of the impact of sarcopenia or myosteatosis will encourage the adoption of routine radiological reporting of body composition and the incorporation of skeletal muscle gauge (SMG) into risk prediction models. This study investigated the prognostic significance of SMG, an aggregate assessment of sarcopenia or myosteatosis, in patients who had undergone an emergency non-trauma-related laparotomy.

**Methods:**

This was a retrospective cohort study of patients aged ≥ 18 years who underwent an emergency laparotomy at the Queen Elizabeth Hospital between January 2014 and December 2020. Body composition and patient outcomes were analysed.

**Results:**

In all, 1090 patients with a mean(standard deviation) age of 62.3(17.5) years underwent emergency laparotomy (bowel obstruction, 52.7%; perforation, 26.3%; ischaemia, 9.5%). Overall 30- and 90-day mortality was 10.0% and 11.6%, respectively. On multivariate analysis, low SMG was associated with worse 30- and 90-day mortality, with odds ratios of 2.12 (95% confidence interval (c.i.) 1.18 to 3.83; *P* = 0.012) and 2.64 (95% c.i. 1.55 to 4.48; *P* < 0.001), respectively. Low SMG was also associated with an increased length of hospital stay (odds ratio 1.45; 95% c.i. 1.22 to 1.72; *P* < 0.001).

**Conclusion:**

A low SMG was associated with increased postoperative mortality and length of hospital stay after emergency laparotomy. Patients undergoing computed tomography imaging for acute abdominal pain should undergo routine reporting of body composition.

## Teaser

Emergency laparotomy is performed for a wide range of life-threatening conditions and is associated with significant morbidity and mortality. This single-centre retrospective study of 1090 patients undergoing emergency laparotomy demonstrates that low skeletal muscle gauge is associated with increased postoperative mortality and length of hospital stay after emergency laparotomy. Patients undergoing computed tomography imaging for acute abdominal pain should undergo routine reporting of body composition.

## Introduction

Emergency laparotomy is performed for a wide range of life-threatening conditions and is associated with significant morbidity and mortality. The UK National Emergency Laparotomy Audit (NELA) prospectively records outcomes for patients undergoing emergency laparotomy to determine mechanisms for service evaluation and improvement. Clinicians and hospitals often focus on outcomes associated with elective care, with significantly lower mortality in this setting. Nonetheless, oesophagectomies and pancreatoduodenectomies are perceived to be potentially the most morbid elective operations performed by surgeons with a background in general surgery training, with mortalities typically at 4.5 and 3%, respectively^1,2^. For the 22 132 emergency laparotomies performed in the UK between December 2020 and November 2021, the in-hospital mortality rate was 9.2%^3^.

Due to the underlying time-critical pathology, patients undergoing emergency laparotomy often have limited opportunity for preoperative work-up, such as echocardiography. Thus, the risk prediction for patients is determined by their current clinical state. Numerous risk prediction models, which have integrated observations and blood tests, can be used to aid clinicians for patient-shared decision-making. The NELA risk model has been developed from the NELA database and has undergone further internal validation^4^. Due to the volume of data now used to model NELA risk prediction, tools such as P-POSSUM^5,6^ and SORT^7^, which use procedural, patient co-morbidity, haemodynamic, and biochemical factors to predict risk, have increasingly become redundant. Risk prediction models enable risk-adjusted benchmarking of patient outcome to facilitate unit improvement. Ongoing validation and the addition of significant pre- and intra-operative determinants of risk are required to improve the accuracy of emergency laparotomy risk prediction.

Measures of skeletal muscle mass and quality, such as myosteatosis (low skeletal muscle radiation attenuation (SMRA)) and sarcopenia (low skeletal muscle index (SMI)), have previously been shown to affect mortality after emergency laparotomy^8–10^. However, body composition measures have not been incorporated into current widely used risk prediction models such as NELA^4^ or the American College of Surgeons National Surgical Quality Improvement Program (NSQIP)^11^ surgical risk predictor.

In studies involving cancer patients^12,13^, it has been reported that patients with both sarcopenia and myosteatosis had worse survival than patients who only had sarcopenia or myosteatosis. These observations raise the possibility that combining SMI and SMRA may have a synergistic effect in predicting patient outcomes. However, the prognostic significance of skeletal muscle gauge (SMG), which is defined as the product of SMI and SMRA, is limited in patients undergoing emergency laparotomy.

The aim of this study was to investigate the prognostic significance of SMG in patients who had undergone an emergency non-trauma-related laparotomy.

## Methods

### Study design and patient selection

All patients over 18 years of age who underwent an emergency laparotomy at the Queen Elizabeth Hospital, Birmingham, between January 2014 and December 2020 were identified from the Trust’s prospectively collected NELA database. To be included, patients were required to have undergone a preoperative computed tomography (CT) scan during their emergency admission as part of their routine care. Patients who underwent a laparotomy secondary to trauma were excluded (further NELA specific exclusion criteria are listed in *Appendix S1*).

### Data collection

Patient demographics, indication for surgery, and clinical data were extracted from a prospectively maintained electronic record database. The NELA-predicted 30-day mortality score was also routinely collected (*Appendix S2*)^14^. The NELA-predicted mortality score incorporates basic patient characteristics, preoperative laboratory tests (creatinine, potassium, sodium, haemoglobin, white blood cell count, and urea), and other clinical measurements, such as heart rate, systolic blood pressure, the Glasgow Coma Scale score, and the UK National Confidential Enquiry into Patient Outcome and Death urgency scale^4^. Expected peritoneal soiling, operative severity, blood loss, and the presence and extent of malignancy are also included in the NELA-predicted mortality score.

Mortality was assessed at 30 and 90 days, and was defined as death due to any cause within 30 and 90 days, respectively, following emergency laparotomy. The length of critical care and hospital stay was prospectively recorded. Prospective data on performance status (Eastern Cooperative Oncology Group (ECOG)/Clinical Frailty Scale) was not universally recorded within the patients’ records and has not been included in the analysis.

### Body composition analysis

Transverse CT images from the third lumbar vertebra (L3) were analysed using SliceOmatic V4.3 software (Tomovision), which enables specific tissue demarcation using Hounsfield unit (HU) thresholds. Skeletal muscle was identified and quantified by a threshold of 29–150 HU^15^. The L3 region contains psoas, erector spinae, quadratus lumborum, transversus abdominus, external and internal obliques, and rectus abdominus muscles. The thresholds used for adipose tissues were −190 to −30 HU for subcutaneous adipose and −150 to −50 HU for visceral adipose^16,17^. Tissue boundaries were manually corrected as needed. Cross-sectional areas (cm^2^) were computed automatically by summing tissue pixels and multiplying by pixel surface area. Cross-sectional area for muscle and adipose tissue was normalized for height (cm^2^/m^2^) and is reported as the skeletal muscle index (SMI), visceral adipose tissue index (VATI), and total adipose tissue index (TATI) at L3.

SMRA was computed as the mean HU value of all pixels included in the skeletal muscle area in the L3 region^18^. SMG was calculated by multiplying the SMI and SMRA, as suggested by Weinberg *et al.*^19^. For simplicity, instead of using units of cm^2^×HU/m^2^ for SMG, an arbitrary unit (a.u.) was used, as in other studies^20,21^. The optimal sex-specific cut-off SMG values were selected on the basis of the association with overall survival of male and female patients in the study cohort using X-tile Version 3.6.1 software (Yale University School of Medicine)^22^. The basic principle of determining the optimal cut-off value was to find the value that produced the largest χ^2^ value in the Mantel–Cox test. Patients were divided into low and high SMG subgroups based on this value.

### Statistical analysis

Data are presented as mean with standard deviation (s.d.) unless otherwise stated.

To determine the utility of the SMI, SMRA, and SMG in predicting clinical outcomes, decision curve analyses were drawn using STATA (version 14.2).

Univariate and multivariate analyses and calculations of odds ratios (ORs) and hazard ratios (HRs) were done using logistic regression and Cox regression models, respectively. Due to the large number of covariates examined, only those that were significant on univariate analysis were included in the multivariate analysis. A backward stepwise procedure was used to derive a final model of significant variables. To remove a variable from the model, the corresponding *P* value had to be > 0.050.

*P* ≤ 0.050 was regarded as statistically significant. Statistical analyses were performed using SPSS^®^ 27.0 (IBM, Armonk, NY, USA).

## Results

### Demographics

In all, 1090 patients underwent an emergency laparotomy during the study period (*Table 1*). The mean(s.d.) age was 62.3(17.5) years and 50.3% of patients (548) were female. The indication for emergency laparotomy was bowel obstruction in 52.7% (574 patients), perforation in 26.3% (287), ischaemia in 9.5% (104), haemorrhage in 3.6% (39), and other causes in 7.9% (86). Body composition was evaluated, and the mean body mass index (BMI) was 26.1(6.2) kg/m^2^. The mean lumbar skeletal muscle index was 43.9(9.5) cm^2^/m^2^, the lumbar VATI was 38.6(33.8) cm^2^/m^2^, the lumbar TATI was 109.3(74.5) cm^2^/m^2^, the SMRA was 27.9(10.8) HU, and the SMG was 1242.0(583.7) a.u. The median NELA-predicted mortality was 4.0% (interquartile range 1.2–10.9%).

**Table 1:** Patient demographics (*n* = 1090)

Age (years), mean(s.d.)	62.3(17.5)
**Sex**	
Male	542 (49.7%)
Female	548 (51.3%)
**Indication for operation**	
Bowel obstruction	574 (52.7%)
Perforation	287(26.3%)
Ischaemia	104(9.5%)
Haemorrhage	39(3.6%)
Other	86(7.9%)
BMI (kg/m^2^), mean(s.d.)	26.1(6.2)
Lumbar SMI (cm^2^/m^2^), mean(s.d.)	43.9(9.5)
Lumbar VATI (cm^2^/m^2^), mean(s.d.)	38.6(33.8)
Lumbar TATI (cm^2^/m^2^), mean(s.d.)	109.3(74.5)
SMRA (HU), mean(s.d.)	27.9(10.8)
SMG (a.u.), mean(s.d.)	1242.0(583.7)
NELA mortality score, median (i.q.r.)	4.0 (1.2–10.9)

Values are *n* (%) unless otherwise stated. s.d., standard deviation; BMI, body mass index; SMI, skeletal muscle index; VATI, visceral adipose tissue index; TATI, total adipose tissue index; SMRA, skeletal muscle radiation attenuation; HU, Hounsfield units; SMG, skeletal muscle gauge; a.u., arbitrary units; NELA, National Emergency Laparotomy Audit; i.q.r., interquartile range.

### Defining the cut-off SMG value

Due to baseline differences in SMG between men and women, sex-specific cut-off values were defined of 1324 and 771 a.u., respectively. In all, 412 (37.8%) patients were allocated into the low SMG group.

### Mortality

Overall 30-day mortality was 10.0% (*Table 2*). On univariate analysis, age, low SMG, and NELA-predicted mortality were significantly associated with mortality. On multivariate analysis both low SMG and NELA-predicted mortality were significantly associated with 30-day mortality after emergency laparotomy.

**Table 2:** Factors associated with 30 day mortality (*n* = 109 of 1090, 10%)

	Univariate analysis		Multivariate analysis	
	Odds ratio	*P*	Odds ratio	*P*
Age	1.04 (1.03, 1.06)	<0.001		0.055
BMI	1.00 (0.97, 1.04)	0.825		
VATI	1.00 (0.99, 1.01)	0.601		
TATI	1.00 (0.99, 1.00)	0.997		
Low SMG	3.84 (2.55, 5.95)	<0.001	2.12 (1.18, 3.83)	0.012
NELA-predicted mortality	1.07 (1.06, 1.10)	<0.001	1.09 (1.05, 1.08)	<0.001

Values in parentheses are 95% confidence intervals. BMI, body mass index; VATI, visceral adipose tissue index; TFI, total fat index; SMG, skeletal muscle gauge; NELA, National Emergency Laparotomy Audit.

Overall 90-day mortality was 11.6% (*Table S1*). On univariate analysis, age (OR 1.04; 95% c.i. 1.03 to 1.05; *P* < 0.001), low SMG (OR 4.21; 95% c.i. 2.83 to 6.27; *P* < 0.001), and NELA-predicted mortality (OR 1.07; 95% c.i. 1.06 to 1.09; *P* < 0.001) were identified as being significantly associated with mortality. On multivariate analysis, both low SMG (OR 2.64; 95% c.i. 1.55 to 4.49; *P* < 0.001) and NELA-predicted mortality (OR 1.06; 95% c.i. 1.05 to 1.08; *P* < 0.001) were significantly associated with 90-day mortality after emergency laparotomy. Age was not significantly associated with mortality on multivariate analysis (*P* = 0.186).

**Fig. 1:**
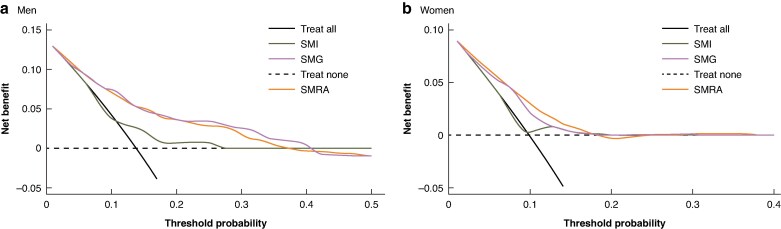
Comparison of decision curves for SMI, SMRA, and SMG in predicting overall survival in a men and b women Dashed lines (treat none) indicate that none of the patients were operated on, and the net benefit was 0; the black lines (treat all) indicate that all the patients were operated on, and the net benefit is represented by the slope of the line. The further the model curves are away from the two reference lines, the better the clinical utility of the factor in predicting overall survival after surgery. SMI, skeletal muscle index; SMRA, skeletal muscle radiation attenuation; SMG, skeletal muscle gauge.

A comparison of decision curves for SMI, SMRA, and SMG examining their impact on overall survival revealed that SMRA and SMG had the greatest clinical utility (*Fig. 1*). This was more evident in male patients.

### Length of hospital stay

The mean overall length of hospital stage was 19.73(±24.77) days (*Table 3*). On univariate analysis, age, BMI, VATI, TATI, low SMG, and NELA-predicted mortality were found to be significantly associated with an increased length of hospital stay. On multivariate analysis, only low SMG and NELA-predicted mortality were significantly associated with an increased overall length of hospital stay after emergency laparotomy.

**Table 3:** Factors associated with the length of hospital stay*

	Univariate analysis		Multivariate analysis	
	Hazard ratio	*P*	Hazard ratio	*P*
Age	1.01 (1.00, 1.01)	<0.001		0.134
BMI	1.02 (1.01, 1.03)	0.005		0.923
VATI	1.00 (1.00, 1.01)	0.014		0.340
TATI	1.00 (1.00, 1.01)	<0.001		0.120
Low SMG	1.80 (1.58, 2.06)	<0.001	1.45 (1.22, 1.72)	<0.001
NELA-predicted mortality	1.03 (1.02, 1.04)	<0.001	1.03 (1.02, 1.04)	<0.001

Values in parentheses are 95% confidence intervals. *The mean(standard deviation) length of hospital stay was 19.73(24.77) days. BMI, body mass index; VATI, visceral adipose tissue index; TFI, total fat index; SMG, skeletal muscle gauge; NELA, National Emergency Laparotomy Audit.

**Fig. 2:**
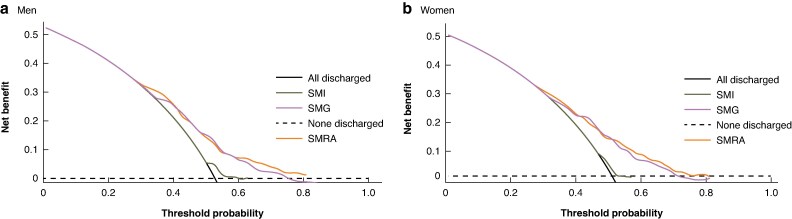
Comparison of decision curves for SMI, SMRA, and SMG in predicting the length of hospital stay in a men and b women Dashed lines indicate the hypothesis that none of the patients will be discharged by Day 14; the black lines indicate the hypothesis that all of the patients will be discharged by Day 14. The further the model curves are away from the two reference lines, the better the clinical utility of the factor in predicting the length of hospital stay after surgery. SMI, skeletal muscle index; SMRA, skeletal muscle radiation attenuation; SMG, skeletal muscle gauge.

A comparison of decision curves for SMI, SMRA, and SMG examining their impact on length of hospital stay revealed that SMRA and SMG had the greatest clinical utility (*Fig. 2*). This was more evident in male patients.

### Length of intensive care unit stay

In all, 40.9% of patients undergoing emergency laparotomy were admitted to intensive care unit (ICU). The mean ICU stay was 6.7(±9.23) days (*Table S2*). On univariate analysis, BMI (OR 1.02; 95% c.i. 1.00 to 1.03; *P* = 0.012), TATI (OR 1.00; 95% c.i. 1.00 to 1.01; *P* < 0.026), low SMG (OR 1.46; 95% c.i. 1.23 to 1.73; *P* < 0.001), and NELA-predicted mortality (OR 1.03; 95% c.i. 1.02 to 1.04; *P* < 0.001) were found to be significantly associated with an increased length of ICU stay. On multivariate analysis, both BMI (OR 1.02; 95% c.i. 1.00 to 1.03; *P* = 0.034) and NELA-predicted mortality (OR 1.03; 95% c.i. 1.02 to 1.04; *P* < 0.001) were significantly associated with an increased overall length of ICU stay after emergency laparotomy. TATI and low SMG were not associated with an increased length of ICU stay on multivariate analysis (*P* = 0.373 and *P* = 0.089, respectively).

## Discussion

This comprehensive assessment of the impact of body composition on emergency laparotomy outcomes confirms that low SMG significantly increases the risk of postoperative mortality. Multivariate analyses demonstrated that SMG has the greatest clinical utility, better than the NELA prediction score. Patients with a low SMG are 2.6-fold more likely to die after emergency laparotomy.

Patients with a low SMG also have a significantly increased length of hospital stay. The clinical impact of SMG is comparable or better than that of other skeletal muscle-related parameters such as SMI and SMRA in patients undergoing an emergency non-trauma-related laparotomy. Combining SMI and SMRA appears to have a synergistic effect on improving prognostic accuracy. Patients undergoing CT imaging for acute abdominal pain should undergo routine reporting of body composition. Predictive models used to determine the risk of postoperative mortality after emergency laparotomy should be updated to incorporate body composition measures such as SMG.

Interestingly, low SMG demonstrated greater clinical relevance in men than in women. This mirrors similar findings in colorectal cancer, where lower abdominal muscle mass levels in men were associated with significantly worse mortality^23,24^. Skeletal muscle mass has also been shown to decline in men after the age of 40 years, whereas it remains more stable in women and only declines slightly in later life^25^. Men have a greater lean mass than women, who have a proportionally higher adipose mass. In the absence of significant lean mass, greater adiposity may provide greater reserve that reduces mortality risk in women undergoing emergency laparotomy.

Over the past few years, measurements of skeletal muscle have been investigated for prognostic or predictive value in patients undergoing emergency surgery^10^. Multimorbidity and frailty have been previously thought to be crucial components to assess when determining suitability for emergency surgery. In UK studies^26^, up to 20% of patients undergoing emergency laparotomy are frail, and subsequent mortality for patients with a clinical frailty score > 5 is threefold higher than that of the general population. However, comparative studies of the predictive significance of both sarcopenia (that is, low SMI) and frailty have determined that sarcopenia is a stronger predictor of 90-day mortality, further confirming the potential benefit it would add to predictive scoring tools^8^. Newer predictive models, such as the Hajibandeh Index, ASA status and Sarcopenia (HAS) score^8^, have evolved to incorporate sarcopenia and have demonstrated improved accuracy compared with the NELA score^27^.

In addition, significant reductions in skeletal muscle quality and structure have been associated with limited postoperative outcomes. Previously identified data from a single UK-based prospective study of body composition analysis after emergency laparotomy^10^ showed that both myosteatosis (that is, low SMRA) and sarcopenia outperform NELA prediction in determining the risk of postoperative mortality after emergency laparotomy.

Recently, Weinberg *et al*.^19^ suggested the integration of SMI and SMRA, and defined the novel integrated metric as SMG. The clinical significance of SMG has so far only been investigated in patients with cancer. A low SMG has been associated with various poor outcome measures in patients with breast, endometrial, ovarian, and colorectal cancer^28,29^. In the present study, a low SMG was shown to be associated with poorer outcomes in patients undergoing non-trauma-related emergency laparotomy.

With respect to risk stratification using SMG, the appropriate cut-off value has not been sufficiently investigated thus far. The best discriminatory values in the present study were 1324 a.u. in men and 771 a.u. in women. Clearly, these cut-off values will need to be evaluated in further studies.

Other scores, such as the American College of Surgeons NSQIP surgical risk predictor, are useful in the clinical work-up of patients undergoing emergency laparotomy, but there is limited external validation for these scores^30–32^. The incorporation of routine sarcopenia assessment has been limited due to the additional time pressures put on radiologists and the limited requests from surgeons. The evolution of artificial intelligence may help reduce some of the future burden body composition analysis will put on radiologists. Community recognition of the value of body composition analysis will support institution acquisition of already available automated software to integrate body composition analysis into day-to-day practice^33^.

This study also identified that low SMG increases the length of hospital stay after emergency laparotomy. The benefits of pre-optimization for surgery have been highlighted in elective care and, despite time pressures, opportunities may exist for emergency patients^34^. All patients admitted to hospital should be assessed for malnutrition. The Malnutrition Universal Screening Tool (MUST) can identify patients at risk of a prolonged hospital stay and inpatient mortality^35^. It may also be used to help assess which patients require nutritional support in the perioperative period. Current guidance advocates that nutritional support may be required in patients with or at risk of malnutrition, such as those who are anticipated not to be able to eat for > 5 days after surgery or those expected not to be able to maintain > 50% of their recommended intake for > 7 days^36^. Routine assessment of body composition on index CT as a supplement to malnutrition scores will facilitate improved prognostication and help determine which patients will require nutritional support. Furthermore, this will identify patients who may require greater inpatient and community physiotherapy intervention and resistance training to reverse the negative effects associated with a low SMG^37^.

This study has a number of strengths. A comprehensive and systematic approach was taken to assess measurements of skeletal muscle to identify whether there is a confirmed clinical correlation with mortality and which measure would be most appropriate to incorporate into future iterations of current predictive models. The study evaluated the impact of body composition on both the total length of hospital stay and ICU stay, which may will enable future infrastructure and workforce planning in the care of emergency surgery patients. NELA data are collected prospectively with outcome data integrated with mortality information from the Office of National Statistics, ensuring high-quality outcome data.

The limitations of the study include it being a single-centre study. In addition, the study included patients undergoing surgery up to 10 years ago, and so there may be variations in outcomes and surgical practices across the duration of the study. Cancer status was not recorded within the database used, and therefore cancer cachexia could not be included in the analysis. Furthermore, due to insufficient numbers, comparative analyses examining indications for surgery and the impact on body composition was not feasible. These limitations all need to be addressed in future studies.

## Supplementary Material

zraf092_Supplementary_Data

## Data Availability

Study data are not routinely available. Applications for data access will be considered on an individual basis. Please contact the corresponding author for further details.

## References

[1] Merath K, Mehta R, Tsilimigras DI, Farooq A, Sahara K, Paredes AZ et al In-hospital mortality following pancreatoduodenectomy: a comprehensive analysis. J Gastrointest Surg 2020;24:1119–1126 31292889 10.1007/s11605-019-04307-9

[2] Oesophago-Gastric Anastomosis Study Group on behalf of the West Midlands Research Collaborative. Rates of anastomotic complications and their management following esophagectomy: results of the Oesophago-Gastric Anastomosis Audit (OGAA). Ann Surg 2022;275:e382–e391 33630459 10.1097/SLA.0000000000004649

[3] National Emergency Laparotomy Audit Project Team. *Eighth Patient Report of the National Emergency Laparotomy Audit*. London: Royal College of Anaesthetists, 2023. https://data.nela.org.uk/information/nelareport8

[4] Eugene N, Oliver CM, Bassett MG, Poulton TE, Kuryba A, Johnston C et al Development and internal validation of a novel risk adjustment model for adult patients undergoing emergency laparotomy surgery: the national emergency laparotomy audit risk model. Br J Anaesth 2018;121:739–748 30236236 10.1016/j.bja.2018.06.026

[5] Copeland GP, Jones D, Walters M. POSSUM: a scoring system for surgical audit. Br J Surg 1991;78:355–360 2021856 10.1002/bjs.1800780327

[6] Mohil RS, Bhatnagar D, Bahadur L, Rajneesh, Dev DK, Magan M. POSSUM and P-POSSUM for risk-adjusted audit of patients undergoing emergency laparotomy. Br J Surg 2004;91:500–503 15048756 10.1002/bjs.4465

[7] Protopapa KL, Simpson JC, Smith NCE, Moonesinghe SR. Development and validation of the surgical outcome risk tool (SORT). Br J Surg 2014;101:1774–1783 25388883 10.1002/bjs.9638 PMC4240514

[8] Hajibandeh S, Hajibandeh S, Hughes I, Mitra K, Puthiyakunnel Saji A, Clayton A et al Development and validation of HAS (Hajibandeh Index, ASA status, Sarcopenia)—a novel model for predicting mortality after emergency laparotomy. Ann Surg 2024;279:501–509 37139796 10.1097/SLA.0000000000005897

[9] Yang TR, Luo K, Deng X, Xu L, Wang RR, Ji P. Effect of sarcopenia in predicting postoperative mortality in emergency laparotomy: a systematic review and meta-analysis. World J Emerg Surg 2022;17:36 35752855 10.1186/s13017-022-00440-0 PMC9233792

[10] Body S, Ligthart MAP, Rahman S, Ward J, May-Miller P, Pucher PH et al Sarcopenia and myosteatosis predict adverse outcomes after emergency laparotomy: a multi-center observational cohort study. Ann Surg 2022;275:1103–1111 33914486 10.1097/SLA.0000000000004781

[11] Bilimoria KY, Liu Y, Paruch JL, Zhou L, Kmiecik TE, Ko CY et al Development and evaluation of the universal ACS NSQIP surgical risk calculator: a decision aid and informed consent tool for patients and surgeons. J Am Coll Surg 2013;217:833–842 24055383 10.1016/j.jamcollsurg.2013.07.385 PMC3805776

[12] Pamoukdjian F, Bouillet T, Lévy V, Soussan M, Zelek L, Paillaud E. Prevalence and predictive value of pre-therapeutic sarcopenia in cancer patients: a systematic review. Clin Nutr 2018;37:1101–1113 28734552 10.1016/j.clnu.2017.07.010

[13] Brown LR, Sayers J, Yule MS, Drake TM, Dolan RD, McMillan DC et al The prognostic impact of pre-treatment cachexia in resectional surgery for oesophagogastric cancer: a meta-analysis and meta-regression. Br J Surg 2023;110:1703–1711 37527401 10.1093/bjs/znad239 PMC10638534

[14] National Emergency Laparotomy Audit Project Team. *Parsimonious NELA Risk Calculator*. https://data.nela.org.uk/riskcalculator/

[15] Mitsiopoulos N, Baumgartner RN, Heymsfield SB, Lyons W, Gallagher D, Ross R. Cadaver validation of skeletal muscle measurement by magnetic resonance imaging and computerized tomography. J Appl Physiol (1985) 1998;85:115–122 9655763 10.1152/jappl.1998.85.1.115

[16] Kvist H, Sjöström L, Tylén U. Adipose tissue volume determinations in women by computed tomography: technical considerations. Int J Obes 1986;10:53–67 3710689

[17] Vehmas T, Kairemo KJ, Taavitsainen MJ. Measuring visceral adipose tissue content from contrast enhanced computed tomography. Int J Obes Relat Metab Disord 1996;20:570–573 8782734

[18] van der Werf A, Dekker IM, Meijerink MR, Wierdsma NJ, de van der Schueren MAE, Langius JAE. Skeletal muscle analyses: agreement between non-contrast and contrast CT scan measurements of skeletal muscle area and mean muscle attenuation. Clin Physiol Funct Imaging 2018;38:366–372 28419687 10.1111/cpf.12422

[19] Weinberg MS, Shachar SS, Muss HB, Deal AM, Popuri K, Yu H et al Beyond sarcopenia: characterization and integration of skeletal muscle quantity and radiodensity in a curable breast cancer population. Breast J 2018;24:278–284 29139618 10.1111/tbj.12952 PMC6414810

[20] Marquardt JP, Roeland EJ, Van Seventer EE, Best TD, Horick NK, Nipp RD et al Percentile-based averaging and skeletal muscle gauge improve body composition analysis: validation at multiple vertebral levels. J Cachexia Sarcopenia Muscle 2021;13:190–202 34729952 10.1002/jcsm.12848 PMC8818648

[21] Zhong Q, Huang JB, Lu J, Xue LW, Lin GT, Xie JW et al Predictive value of a new muscle parameter in patients with resectable gastric cancer: a pooled analysis of three prospective trials. Ann Surg Oncol 2024;31:3005–3016 38270825 10.1245/s10434-024-14913-w PMC10997550

[22] Camp RL, Dolled-Filhart M, Rimm DL. X-tile: a new bio-informatics tool for biomarker assessment and outcome-based cut-point optimization. Clin Cancer Res 2004;10:7252–7259 15534099 10.1158/1078-0432.CCR-04-0713

[23] Caan BJ, Meyerhardt JA, Kroenke CH, Alexeeff S, Xiao J, Weltzien E et al Explaining the obesity paradox: the association between body composition and colorectal cancer survival (C-SCANS Study). Cancer Epidemiol Biomarkers Prev 2017;26:1008–1015 28506965 10.1158/1055-9965.EPI-17-0200 PMC5647152

[24] van Baar H, Winkels RM, Brouwer JGM, Posthuma L, Bours MJL, Weijenberg MP et al Associations of abdominal skeletal muscle mass, fat mass, and mortality among men and women with stage I–III colorectal cancer. Cancer Epidemiol Biomarkers Prev 2021;29:956–965 10.1158/1055-9965.EPI-19-1134 32132148

[25] Lee MM, Jebb SA, Oke J, Piernas C. Reference values for skeletal muscle mass and fat mass measured by bioelectrical impedance in 390 565 UK adults. J Cachexia Sarcopenia Muscle 2020;11:487–496 31943835 10.1002/jcsm.12523 PMC7113534

[26] Parmar KL, Law J, Carter B, Hewitt J, Boyle JM, Casey P et al Frailty in older patients undergoing emergency laparotomy: results from the UK observational Emergency Laparotomy and Frailty (ELF) study. Ann Surg 2021;273:709–718 31188201 10.1097/SLA.0000000000003402

[27] Linganathan S, Hughes I, Puthiyakunnel Saji A, Mitra K, Hajibandeh S, Hajibandeh S. HAS (Hajibandeh Index, American Society of Anesthesiologists status, and Sarcopenia) model *versus* NELA (National Emergency Laparotomy Audit) score in predicting the risk of mortality after emergency laparotomy: a retrospective cohort study. Cureus 2023;15:e50180 38077684 10.7759/cureus.50180 PMC10706199

[28] Polen-De C, Fadadu P, Weaver AL, Moynagh M, Takahashi N, Jatoi A et al Quality is more important than quantity: pre-operative sarcopenia is associated with poor survival in advanced ovarian cancer. Int J Gynecol Cancer 2022;32:1289–1296 35680140 10.1136/ijgc-2022-003387

[29] Park IK, Yang SS, Chung E, Cho ES, Lee HS, Shin SJ et al Skeletal muscle gauge as a prognostic factor in patients with colorectal cancer. Cancer Med 2021;10:8451–8461 34643052 10.1002/cam4.4354 PMC8633260

[30] Thahir A, Pinto-Lopes R, Madenlidou S, Daby L, Halahakoon C. Mortality risk scoring in emergency general surgery: are we using the best tool? J Perioper Pract 2021;31:153–158 32368947 10.1177/1750458920920133

[31] Barghash M, Iskandar A, Fawzy SI, Effiom D, Huck C, Hajibandeh S et al Predictive performance of NELA *versus* P-POSSUM mortality scores: are we underestimating the risk of mortality following emergency laparotomy? Cureus 2022;14:e32859 36694527 10.7759/cureus.32859 PMC9867845

[32] Hunter Emergency Laparotomy Collaborator Group . High-risk emergency laparotomy in Australia: comparing NELA, P-POSSUM, and ACS-NSQIP calculators. J Surg Res 2020;246:300–304 31648068 10.1016/j.jss.2019.09.024

[33] Sudarsanam A, Davies AH, Rockall A, Sekhon H, Salim S. A fully automated sarcopenia segmentation tool using artificial intelligence can reliably assist in preoperative evaluation of patients undergoing major vascular surgery. J Vasc Surg 2024;79:e235–e236

[34] Poulton T, Murray D; National Emergency Laparotomy Audit (NELA) Project Team. Pre-optimisation of patients undergoing emergency laparotomy: a review of best practice. Anaesthesia 2019;74:100–107 10.1111/anae.14514 30604422

[35] Aloy Dos Santos T, Luft VC, Souza GC, de Albuquerque Santos Z, Keller Jochims AM, Carnevale de Almeida J. Malnutrition Screening Tool and Malnutrition Universal Screening Tool as a predictors of prolonged hospital stay and hospital mortality: a cohort study. Clin Nutr ESPEN 2023;54:430–435 36963890 10.1016/j.clnesp.2023.02.008

[36] Weimann A, Braga M, Carli F, Higashiguchi T, Hübner M, Klek S et al ESPEN practical guideline: clinical nutrition in surgery. Clin Nutr 2021;40:4745–4761 34242915 10.1016/j.clnu.2021.03.031

[37] Supriya R, Singh KP, Gao Y, Gu Y, Baker JS. Effect of exercise on secondary sarcopenia: a comprehensive literature review. Biology (Basel) 2022;11:51 10.3390/biology11010051 PMC8773430 35053049

