# Supplementary material for: Evaluation of sarcopenia and myosteatosis to determine the impact on mortality after emergency laparotomy
Source: BJS Open. 2025 Aug 5;9(4):zraf092. doi: 10.1093/bjsopen/zraf092 (PMC12341672; doi:10.1093/bjsopen/zraf092)
Supplement: zraf092_Supplementary_Data [file zraf092_supplementary_data.docx]

**Evaluation of Sarcopenia and Myosteatosis to determine the Impact on Mortality after Emergency Laparotomy**

Richard PT Evans^1,2^, Dimit Raveshia^1^, Mei Sien Liew^1^, Anna Jackowski^1^, Aaron Kisiel^1^, Ewen A Griffiths^1,2^, Benjamin HL Tan^1,2^

1. Department of Upper GI Surgery, Queen Elizabeth Hospital, Birmingham, UK.
2. Institute of Immunology and Immunotherapy, University of Birmingham, UK.

**Corresponding Author:**

Mr. Benjamin Tan

Consultant Upper GI Surgeon,

Department of Upper Gastrointestinal Surgery,

Area 6, 7th Floor, Queen Elizabeth Hospital Birmingham,

Mindelsohn Way, Edgbaston, Birmingham

B15 2WB

Email: benjamin.tan@uhb.nhs.uk

Telephone: 0121 3715886

**Supplementary Materials - Index**

| **Supplementary Appendixes** |  |
| --- | --- |
| NELA inclusion/exclusion criteria | *page 3* |
| NELA Pre-Op Risk Stratification | *page 4* |
| **Supplementary Figures and Tables** |  |
| Supplementary Table 1 Factors associated with 90 Day Mortality | *page 5* |
| Supplementary Table 2 Factors associated with length of ITU stay | *page 6* |
| **References** | *page 7* |
|  |  |

**Supplementary Appendixes**

**NELA Inclusion/Exclusion Criteria**^1^

1. Patients under 18

2. Elective laparotomy / laparoscopy

3. Diagnostic laparotomy/laparoscopy where no subsequent procedure is performed (NB, if no procedure is performed because of inoperable pathology, then include)

4. Appendicectomy +/- drainage of localised collection unless the procedure is incidental to a non-elective procedure on the GI tract

5. Cholecystectomy +/- drainage of localised collection unless the procedure is incidental to a non-elective procedure on the GI tract

(All surgery involving the appendix or gallbladder, including any surgery relating to complications such as abscess or bile leak is excluded. The only exception to this is if carried out as an incidental procedure to a more major procedure. We acknowledge that there might be extreme cases of peritoneal contamination, but total exclusion avoids subjective judgement calls about severity of contamination.)

6. Non-elective hernia repair without bowel resection or division of adhesions

7. Minor abdominal wound dehiscence unless this causes bowel complications requiring resection

8. Non-elective formation of a colostomy or ileostomy as either a trephine or a laparoscopic procedure (NB: if a midline laparotomy is performed, with the primary procedure being formation of a stoma then this should be included)

9. Vascular surgery, including abdominal aortic aneurysm repair

10. Caesarean section or obstetric laparotomies

11. Gynaecological laparotomy (but see comment above about inclusion of gynae-oncology)

12. Ruptured ectopic pregnancy, or pelvic abscesses due to pelvic inflammatory disease

13. Laparotomy/laparoscopy for pathology caused by blunt or penetrating trauma

14. All surgery relating to organ transplantation (including returns to theatre for any reason following transplant surgery)

15. Surgery relating to sclerosing peritonitis

16. Surgery for removal of dialysis catheters

17. Laparotomy/laparoscopy for oesophageal pathology

18. Laparotomy/laparoscopy for pathology of the spleen, renal tract, kidneys, liver, gall bladder and biliary tree, pancreas or urinary tract

19. Returns to theatre for complications (eg bowel injury, haematoma, collection) following non-GI surgery are excluded i.e., returns to theatre following renal, urological, gynaecological, vascular, hepatic, pancreatic, splenic surgery are excluded. Specific exceptions to this list are complications requiring the assistance of a general surgeon following an interventional radiology procedure; or following gynaecology-oncology surgery – these cases should now be INCLUDED, as per inclusion criteria above).

**NELA Pre-Op Risk Stratification**^2^

**Age**

**Sex**

**Diabetes**

**Most recent blood glucose**

**ASA**

**Creatinine**

**Lactate**

**Albumin**

**Urea**

**WCC**

**Pulse rate**

**Systolic blood pressure**

**Glasgow Coma Scale**

**Patient’s respiratory history and chest x-ray appearance** (no dyspnoea, dyspnoea on exertion or CXR shows mild COAD, dyspnoea limiing exertion to <1 flight or CXR shows moderate COAD, dyspnoea at rest/rate >30 at rest or CXR shows fibrosis or consolidation)

**Likely degree of peritoneal soiling** (none, serous fluid, localised pus, free pus/blood/bowel content)

**Severity of malignancy** (none, primary only, nodal mets, distant mets)

**What was the global impression of theatre access for surgery at the time of booking the case?** (expedited >18 hours, urgent 6-18 hours, urgent 2-6 hours, immediate < 2hours)

**Indication for surgery** (bleeding, obstruction, sepsis, ischaemia, other)

**Supplementary Figures and Tables**

Supplementary Table 1

Factors associated with 90 Day Mortality (n=126/1090, 11.6%)

|  | Univariate Analysis | | | Multivariate analysis | | |
| --- | --- | --- | --- | --- | --- | --- |
|  | Odds Ratio | 95% CI | *P* | Odds Ratio | 95% CI | *P* |
| Age | 1.04 | 1.03 – 1.05 | <0.001 |  |  | 0.186 |
| BMI (kg/m^2^) | 1.01 | 0.98 – 1.04 | 0.585 |  |  |  |
| VATI (cm^2^/m^2^) | 1.00 | 0.99 – 1.01 | 0.301 |  |  |  |
| TATI (cm^2^/m^2^) | 1.00 | 0.99 – 1.01 | 0.681 |  |  |  |
| Low SMG | 4.21 | 2.83 – 6.27 | <0.001 | 2.64 | 1.55 – 4.48 | <0.001 |
| NELA predicted mortality | 1.07 | 1.06 – 1.09 | <0.001 | 1.06 | 1.05 – 1.08 | <0.001 |

Abbreviations: 95% CI, 95% Confidence Interval. BMI, Body mass index. VATI, Visceral adipose tissue index. TFI, Total fat index. SMG, Skeletal muscle gauge. NELA, National emergency laparotomy audit.

Supplementary Table 2

Factors associated with length of ITU stay (n= 446)

(mean: 6.7 ± 9.23 days)

|  | Univariate Analysis | | | Multivariate analysis | | |
| --- | --- | --- | --- | --- | --- | --- |
|  | Hazard Ratio | 95% CI | *P* | Hazard Ratio | 95% CI | *P* |
| Age | 0.99 | 0.99 – 1.01 | 0.700 |  |  |  |
| BMI (kg/m^2^) | 1.02 | 1.00 – 1.03 | 0.012 | 1.02 | 1.00 – 1.03 | 0.034 |
| VATI (cm^2^/m^2^) | 1.00 | 0.99 – 1.01 | 0.169 |  |  |  |
| TATI (cm^2^/m^2^) | 1.00 | 1.00 – 1.01 | 0.026 |  |  | 0.373 |
| Low SMG | 1.48 | 1.23 – 1.73 | <0.001 |  |  | 0.089 |
| NELA predicted mortality | 1.03 | 1.02 – 1.04 | <0.001 | 1.03 | 1.02 – 1.04 | <0.001 |

Abbreviations: 95% CI, 95% Confidence Interval. BMI, Body mass index. VATI, Visceral adipose tissue index. TFI, Total fat index. SMG, Skeletal muscle gauge. NELA, National emergency laparotomy audit.

**References**

1. NEAL Inclusion/Exclusion. *https://data.nela.org.uk/information/nelaincexl*.

2. NELA Calculator. *http://data.nela/org.uk/*.
